# Radical Hemiscrotectomy and En Bloc Orchidectomy: Surgical Technique and Perioperative and Oncologic Outcomes of a Supra-Regional UK Referral Centre

**DOI:** 10.1245/s10434-021-10315-4

**Published:** 2021-07-16

**Authors:** Christian Daniel Fankhauser, Allaudin Issa, Esther W. C. Lee, Christoph Oing, Pedro Oliveira, Arie Parnham, Jeremy Oates, Vijay Sangar, Aziz Gulamhusein, Noel Clarke

**Affiliations:** 1 Department of Urology, The Christie NHS Foundation Trusts, Manchester, UK; 2 Department of Medical Oncology, The Christie NHS Foundation Trust, Manchester, UK; 3 Department of Oncology, Hematology and Bone Marrow Transplantation with Division of Pneumology, University Medical Center Eppendorf, Hamburg, Germany; 4 Manchester University NHS Foundation Trust, Manchester, UK; 5 Manchester Academic Health Sciences Centre, Manchester, UK; 6 University of Manchester, Manchester, UK; 7 The Salford Royal NHS Foundation Trust, Manchester, UK

## Abstract

**Background and Purpose:**

Hemiscrotectomy with en bloc orchidectomy represents a radical primary, completion, or salvage option in men with inguinoscrotal cancers. We describe our surgical technique and peri-operative and oncological outcomes.

**Patients and Methods:**

Retrospective cohort study of 16 men treated at a supra-regional referral centre with open radical hemiscrotectomy with or without en bloc orchidectomy between 2010 and 2020. Peri-operative and survival outcomes were analysed.

**Results:**

Radical hemiscrotectomy with or without en bloc orchidectomy was performed on 16 patients comprising 7 well-differentiated liposarcomas, 4 dedifferentiated liposarcomas, 2 leiomyosarcomas, 1 mesothelioma, 1 rhabdomyosarcoma and 1 mammary type myofibroblastoma. Primary hemiscrotectomy was performed in four, completion hemiscrotectomy in nine and salvage hemiscrotectomy in three. The median hospital stay was 2 days [interquartile range (IQR) 2–4]. Four patients (25%) had post-operative complications including wound infection or haematoma. During a median follow-up of 18 months (IQR 2–66), one patient (6%) died following a recurrence in the pelvis and retroperitoneum.

**Discussion:**

and Conclusions

If careful dissection is performed, radical hemiscrotectomy and en bloc orchidectomy is a radical but safe procedure with a short hospital stay. Haematoma and infection represent the main complications, and within limited follow-up most men showed no recurrence.

**Supplementary Information:**

The online version contains supplementary material available at 10.1245/s10434-021-10315-4.

Several rare urogenital cancers arising from tissues of the spermatic cord, epididymis, testis or scrotal skin have a high risk of local recurrence. A radical resection by a hemiscrotectomy with or without en bloc orchidectomy is therefore recommended to try to reduce this complication. Given the limited literature describing this surgery, we summarised our surgical technique and peri-operative and oncological outcomes.

## Methods

### Pre-operative Staging

All men with a suspected non-germ cell/non-penile primary inguinoscrotal cancer are staged with computerised tomography of the chest and abdomen to assess the most common sites of metastatic disease. If involvement of adjacent structures including the penis, urethra, femoral vessels or pelvic floor is suspected, we recommend further staging with magnetic resonance imaging.

### Informed Consent

Patients are counselled about their diagnosis, management options and prognosis by medical and specialist nurse practitioners. Prior to surgery, expected benefits, likelihood of success and risks of complications and recurrence are discussed. Potential surgical risks pertinent to this particular surgery include bleeding, haematoma, infection, acute and chronic pain, wound breakdown, inguinal hernia, injury to nerves causing neuropathy, urethral/vascular damage, cosmesis, small scrotum, displaced contralateral testis, lypmhoedema of penis/scrotum and inability to resect completely heightening the risk of cancer recurrence locally and distally.

### Surgical Procedure

#### Patient Positioning and Approach

**Fig. 1:**
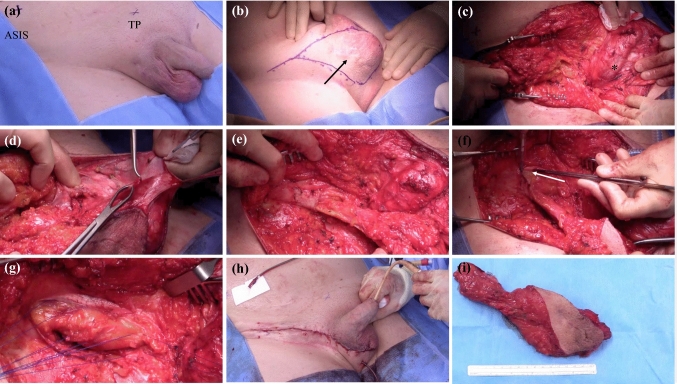
Important surgical steps during hemiscrotectomy with en bloc orchidectomy include identification of key anatomical landmarks including (**a**) the anterior superior iliac spine (ASIS), inguinal ligament, pubic tubercle (PT) as well as the penile and scrotal raphe, and superficial skin incision from the the lateral third of the inguinal ligament to the scrotal raphe and back in a tear drop configuration. This should incorporate any previous incisional scars (black arrow) (**b**), exposure of the inguinal ligament, the spermatic cord, Buck’s facia of penis and the urethra (*) (**c**), identification of the scrotal septum and resection if needed (**d**), completion of en bloc orchidectomy between dartos and external spermatic fascia up to the external inguinal ring (**e**), opening of external oblique fascia in the direction of its fibres (white arrow) towards the internal inguinal ring (**f**), exposure of the internal ring until preperitoneal fat is identified, approximation of the conjoint tendon to the inguinal ligament (**g**), placement of two 14 French suctions, closure of the dead space and subcuticular wound closure (**h**). Specialist histopathological analysis (**i**)

The patient is positioned supine on the operating table with legs slightly abducted. Drapes are placed allowing for visualisation of important anatomical landmarks including the anterior superior iliac spine (ASIS), inguinal ligament, pubic tubercle, and the penis and scrotum to the perineum (Fig. 1a). In some cases, patients may need to be positioned in Lloyd Davis (lithotomy) to improve access to the scrotum. A urinary catheter is placed to facilitate intra-operative recognition of the urethra and to allow peri-operative bladder drainage. The patient is given one intravenous dose of prophylactic antibiotics (co-amoxiclav).

#### Scrotal Resection

A superficial skin incision in a ‘tear drop’ configuration is marked with pen and performed from the the lateral third of the inguinal ligament to the scrotum, including the raphe (Fig. 1b). Any previous incisional scars from previous surgery within the inguinoscrotal region should be resected, including tissue potentially contaminated with cancer cells. The incision is deepened down to the inguinal ligament and is continued medially around the spermatic cord further to Buck’s facia of the penis, where care should be taken not to injure the urethra (Fig. 1c). From the base of the penis, the plane of the scrotal septum can be followed; however, it is often included in the resection to ensure oncological control (Fig. 1d). Care should be taken not to dissect too far caudal and lateral to avoid injury to the long saphenous and femoral veins. An en bloc orchidectomy is performed with the overlying scrotal skin attached (Fig. 1e). The proximal extent of the inguinal cord resection is predicated on the pre-operative characteristic of the tumour assessed clinically and radiologically. If necessary, the inguinal canal may need exploration, with resection back to the internal ring as described below.

#### Resection of the Spermatic Chord and Reconstruction

The en bloc orchidectomy specimen is lifted, and the external oblique fascia is opened in the direction of its fibres towards the internal inguinal ring (Fig. 1f). The cremaster muscle fibres are separated perpendicular to the cord to expose the internal ring until preperitoneal fat is identified. The spermatic cord is clamped, transfixed and divided, ensuring the specimen is fully excised. In rare cases with intra-abdominal extension of tumour, a laparotomy is required to ensure complete resection. The inguinal canal is supported in a Bassini fashion by approximating the conjoint tendon (formed by the distal ends of the transversus abdominis and internal oblique muscles) to the inguinal ligament with interrupted 3-0 polypropylene, and the oblique fascia is closed using a running 3-0 polypropylene (Fig. 1g).

#### Drains, Closure and Dressings

Two 14 French suction drains are placed (scrotal and inguinal), and the dead space is closed with 2-0 and 3-0 polyglactin sutures, a subcuticular wound closure with a 3-0 monofilament synthetic absorbable suture (Fig. 1h). The wound is covered with skin glue, film dressing spray or adhesive film dressings. Gentle pressure is applied to the wound using a suspensory scrotal support in the immediate post-operative period.

#### Postoperative Care

**Fig. 2:**
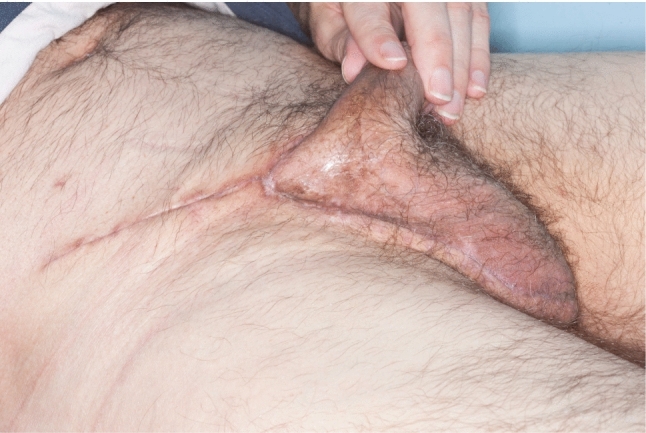
Inguinoscrotal scar 1 month after hemiscrotectomy and residual spermatic cord resection

The patient is encouraged to mobilise, eat and drink immediately after surgery. No post-operative antibiotic prophylaxis is required, and peri-operative thromboprophylaxis using low-dose low-molecular-weight heparin is commenced immediately after surgery. Drains can be removed on post-operative day 1 if the drain volume is below 50 mL/24 h. Early follow-up in a urooncology outpatient clinic for wound inspection and to rule out inguinal hernia is recommended (Fig. 2).

#### Cohort Study and Video

The patients consented to the use of both photographic and videographic material for education in accordance with local institutional guidelines. Data were collected retrospectively for patients identified from surgical theatre diaries between 2010 and 2020. Local recurrence-free, cancer-specific and overall survival as well as peri-operative and post-operative complications were extracted from medical charts by a board-certified urologist. Primary resection was defined as hemiscrotectomy without any prior surgery of the scrotum. Completion surgery was defined as hemiscrotectomy after prior scrotal surgery such as surgical biopsy, hydrocelectomy or orchidectomy with the aim to ensure complete resection of either positive surgical margin or aggressive histology. Salvage surgery was defined as hemiscrotectomy to treat macroscopic recurrence after prior scrotal surgery.

### Statistical Analysis

Descriptive statistics are used to report patient and tumour characteristics as well as peri-operative and oncological outcomes. Statistical analyses were performed using R version 3.1.3 (R Foundation for Statistical Computing, Vienna, Austria).

## Results

Radical hemiscrotectomy with or without en bloc orchidectomy was performed in 16 patients with a mean age of 56 years (range 20–80 years). Race/ethnicity distribution was not reported in the electronical health records. Four men had a primary resection and nine underwent completion surgery, with the remaining three undergoing salvage surgery. In men with primary surgery, two patients had well-differentiated liposarcoma (WDLS), one rhabdomyosarcoma and one mammary type myofibroblastoma. The patient with rhabdomyosarcoma underwent primary surgery after initial biopsy and neoadjuvant chemotherapy with ifosfamid, vincristine and dactinomycin. He subsequently received adjuvant chemotherapy with the same regimen and radiotherapy with 50.4 Gy in 28 fractions to the retroperitoneal lymph nodes and maintenance vinorelbine and cyclophosphamide.

**Fig. 3:**
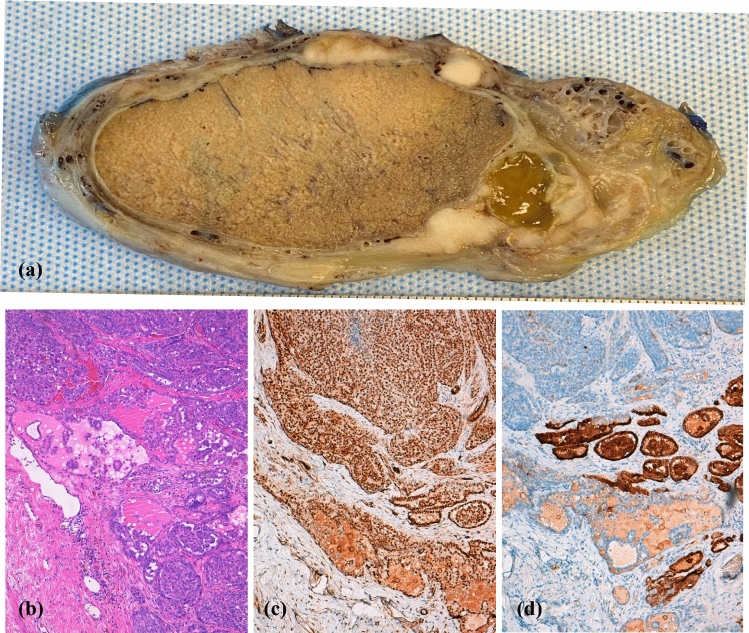
Hemiscrotectomy with en bloc orchidectomy for mesothelioma of the tunica vaginalis testis with multifocal involvement (**a**). This specimen represents an epithelial subtype with microscopy at 100× magnification using the following stains: haematoxylin and eosin (**b**), WT1 immunohistochemistry (**c**) and calretinin immunocytochemistry (**d**)

Completion surgery was performed within a mean of 3 months (range 1–4 months) after prior testicular surgery (inguinal/testicular orchidectomy or hydrocelectomy). Histological diagnoses included dedifferentiated liposarcoma (DLS) in four, WDLS in two, leiomyosarcoma in two and mesothelioma in one patient. In the subsequent hemiscrotectomy specimens, six out of nine (67%) showed no evidence of tumour, whereas three patients had residual disease including LDS, WDLS and mesothelioma (Fig. 3). Salvage surgery was performed in two patients 31 and 50 months after inguinal excision of a lipomatous mass which was incorrectly diagnosed as lipoma and reclassified on review as WDLS. The third patient with salvage surgery was operated 72 months after orchidectomy as the patient initially refused completion orchidectomy and failed to re-attend thereafter.

The median hospital stay was 2 days (IQR 2–4 days). Four patients (25%) had 90-day post-operative complications. Two (12%) had wound infections requiring oral antibiotics (Clavien–Dindo II), with the remaining two (12%) developing a haematoma (Clavien–Dindo I) which was managed conservatively.

During a median follow-up of 18 months (IQR 2–66), one patient (6%) initially presenting with locally advanced dedifferentiated liposarcoma measuring 130 × 50 × 48 mm had recurrence. Metastatic disease in the pelvic and retroperitoneal lymph nodes was diagnosed 2 months after hemiscrotectomy with rapid clinical deterioration. He died 4 months after initial diagnosis despite palliative chemotherapy with doxorubicin and olaratumab.

## Discussion

This report summarises important pre-, intra- and post-operative steps and reports peri-operative and oncological outcomes mainly relating to inguinoscrotal sarcoma.

In the pre-operative setting, imaging is vital for pre-operative planning and for help with appropriate patient counselling about the extent of resection. Consideration is also given to whether a biopsy is required and whether this may alter management or extent of resection. For example, cancers limited to the scrotal skin itself do not require en bloc orchidectomy, and if there is not sufficient skin cover on the ipsilateral side, a transposition of the testis to the scrotum on the contralateral side can be considered^1^ or ectopic placement in the thigh. In such cases, skin flaps for reconstruction are possible but should only be offered after appropriate counselling of the patients that changes of flaps may mimic local recurrence and trigger unnecessary biopsies or reinterventions during follow-up.^2^ Additionally, pre-operative imaging using magnetic resonance imaging helps to identify and adequately plan surgery in men with locally advanced disease which may may require a multidisciplinary surgical approach to allow e.g. reconstruction of vessels or the inguinal ligament.

Important intraoperative steps include excision of previous scars, resection of the testicular septum and complete exposure of the internal inguinal ring. Excision of previous scars is required owing to the risk of tumour contamination from previous incomplete resection. The scrotal septum should be resected if there is risk of a positive surgical margin (especially in instances of sarcoma), particularly as there is no significant added morbidity associated with this. Similarly, removal of any spermatic cord remnant should be performed as cephalad as possible to the internal inguinal ring. Closure and reconstruction of the inguinal canal represent important steps to prevent inguinal hernias. In tumours invading the inguinal canal structures, resection and reconstruction of the inguinal ligament preferentially with a pedicled fascia lata flap^3^ instead of a prosthetic mesh^4^,^5^ should be considered.

Given this study is a retrospective single-centre surgical review, it has significant limitations. Inevitably, there was some selection bias in our series, as small inguinoscrotal cancers are often treated in general urology departments with scrotal-sparing surgery with or without orchidectomy. Patients with rare, large or sarcomatous tumours, or with tumour recurrence after primary surgery were referred to our institution. The study also has insufficient statistical power to comment on cancer-related outcome. Hemiscrotectomy with or without en bloc orchidectomy represents an important surgical procedure for several indications including sarcomas of the inguinal cord,^6^–^8^ mesotheliomas of the tunica vaginalis testis,^9^ locally advanced or recurring germ cell tumours^10^–^12^ as well as for basal or squamous cell carcinoma, melanoma or extramammary Paget’s disease of the scrotum.^13^ However, stringent indication for hemiscrotectomy with or without en bloc orchidectomy instead of less radical options including wide local excision or orchidectomy only is not defined in the current literature, but our group is working on systematic reviews and meta-analysis with the aim to answer this question.^6^

The primary goal of this report is to illustrate the surgical technique, potential complications and early oncological outcomes. Given the rarity of inguinoscrotal cancers requiring hemiscrotectomy, it is unlikely that larger prospective studies will be performed.

## Conclusions

Sarcomas and other rare inguinoscrotal tumours are rare, usually requiring radical primary resection. Given their rarity, this is best performed in a tertiary referral centre, where there is likely to be greater expertise in the joint multi-disciplinary management of patients of this type. If careful dissection is performed, radical hemiscrotectomy and en bloc orchidectomy is a radical but safe procedure with a short hospital stay. Haematoma and infection represent the main complications, and within limited follow-up most men showed no recurrence.

## Supplementary Information

Below is the link to the electronic supplementary material.

Supplementary file1 (MP4 71096 KB)
